# What Is the Amount of China’s Building Floor Space from 1996 to 2014?

**DOI:** 10.3390/ijerph17165967

**Published:** 2020-08-17

**Authors:** Linwei Pan, Minglei Zhu, Ningning Lang, Tengfei Huo

**Affiliations:** 1 School of Economics and Management, Chongqing Jiaotong University, Chongqing 400074, China; 990201200037@cqjtu.edu.cn; 2 Research Center for Economy of Upper Reaches of the Yangtse River, Chongqing Technology and Business University, Chongqing 400067, China; zhuml@amc023.com; 3 Chongqing Yukang Asset Management Co., Ltd., Chongqing 400020, China; 4 School of Management Science and Real Estate, Chongqing University, Chongqing 400044, China; langningn@163.com; 5 School of Economics and Management, Hebei University of Technology, Tianjin 300401, China

**Keywords:** China, building stock, energy consumption in the building sector, residential building, commercial building

## Abstract

The amount of building floor space (BFS) plays a key role in the energy and material demand prediction. Unfortunately, BFS estimation has faced the challenge of ineffective and inadequate approaches, and thus reliable data concerning China’s BFS is unavailable. This study proposes a new estimation method for China’s BFS and then estimates historical BFS by type in China from 1996 to 2014. The results show that total Chinese BFS grew from 28.1 billion m^2^ in 1996 to 61.3 billion m^2^ in 2014, increasing more than twice, with an annual growth rate of 4.4% from 1996 to 2014. During 1996–2014, urban residential BFS witnessed the highest annual increase rate (9.3%), while the growth rate for commercial and rural residential BFS was lower: 4.4% and 1.6%, respectively. By comparing with available statistics data, this study finds the model deviations are well below 5%, which indicates the reliability of the proposed method and robustness of the results. The proposed method not only can address the deficiencies of statistic yearbook and overcome the shortages of previous estimation approaches but also can derive more accurate and reliable data. This study lays a sound basis for the following study on building stock and building energy efficiency work.

## 1. Introduction

The building sector consumes a great amount of energy and resources, and it is an important contributor to the global issues caused by greenhouse gas emissions [1]. In most developed countries, the building sector consumes approximately more than one-third of the final energy use [2,3,4,5]. In 2014, China’s building energy consumption was up to 814 million tons of standard coal, which was 1.7 times that in 2000 accounting about 20% in the total energy consumption [6], and this proportion probably grew to 35% by 2020 [7,8,9]. The main driving force for the growth of building energy demand is the huge volume of the existing building stocks (represented by building floor space (BFS)) [10,11]. With the gradual deepening of urbanization process, there is a trend that more commercial energy will be consumed in China’s building sector, and more carbon emissions will be emitted from those fossil fuels simultaneously in the future. Thereby, the building floor space plays a decisive role in the future energy demand.

Besides, the magnitude of the building stock is of vital importance to the urban renewal decision making. By 2019, China’s urbanization rate has increased dramatically by 39.5% compared to 1980, and it is expected to rise to approximately 80% by 2050 [12,13]. Behind the rapid increase of urbanization rate is the influx of massive migrant workers [14,15,16]. To accommodate new migrants in urban areas, China is now engaged in construction activity on the largest scale in the world [17,18,19]. Due to the rapid advance of urbanization, people are pursuing a higher quality of life [20]. This results in an increased demand for new housing construction, and a large volume of building projects are constructed [21,22,23]. Urban sprawl and urban renewal strategies are usually implemented to satisfy those demands, through which considerable demolition and reconstruction works have been carried out. Thus, the size of the building stock is of great significance to decide how many new buildings need to be constructed and how many buildings need to be demolished.

Moreover, the volume of the building stock has significant implications on the material flow in the building sector. China’s new building floor area per year is about 2 billion m^2^, accounting for nearly half of the world’s new building area [24], and 30–40% of the world’s annual output of cement and steel is therefore consumed [25]. About 9% of total energy and 15% of industrial energy would be consumed for generating these building materials [26,27,28]. Besides, the shorter lifespan of the existing buildings in China also leads to the fast growth of construction activities, and this would directly result in massive building wastes [29]. The shorter lifespan of the existing Chinese building stock also leads to the high new construction rate, and this will further result in the material and energy demand. Therefore, accurately measuring the volume of the building floor space is vital for quantifying the material flows.

In recent years, research on the BFS has been mainly associated with the prediction of future energy consumption and material demand [7,30,31,32,33,34,35,36,37,38]. The base year BFS was directly obtained in their studies from China Statistical Yearbook (*CSY*). In fact, such processing methods are highly problematic, due to there being many deficiencies in the current statistical system in China, which hinders the research on building energy efficiency and plan. To cover the research gap, this study aims to propose a new estimation method for China’s building floor space and predict the time-series data of the BFS by type from 1996 to 2014. The proposed method can eliminate the deficiencies of inconsistent statistical caliber and can derive reliable and robust BFS results. The results can also provide reliable data support for the government to devise reasonable measures and promote the building energy-saving work.

The rest of this study is presented as follows. The next section is the literature review, and the following section is the systematic analysis in the existing deficiencies of statistical indicators regarding BFS in the Statistical Yearbook. After this, this study provides details on the estimation methodology. Then, the estimation results are presented. The subsequent section is discussion and analysis of the results, as well as the reliability analysis. The final section is the conclusion and future directions for this study.

## 2. Literature Review

Scholars have paid considerable attention to building stock due to its significance to building energy consumption and resource demand projection. As for the building floor space in other countries, scholars adopted some methods, such as dynamic material flow analysis (MFA) [39], synthetic model [40], dynamic stock-driven model [41], etc. Regarding China’s building floor space, Hu et al. estimated and predicted the dwelling stock of China from 1900 to 2100 adopting MFA method [36,42]. In Hu et al.’s study, they obtained the urban residential stock through multiplying the urban per capita BFS by the urban population, and the same approach is used in the rural residential stock. Besides, they used the background flows (such as birth/death rate, internal migration flows from rural to urban) to check the data. It is obvious that their data processing method is also problematic as the per capita floor space of buildings in *CSY* does not include the urban collective households but only covers the family households, which would cause overestimation the magnitudes of building floor space. Besides, they did not identify the size of commercial BFS, which is an important component in China’s building stock. In addition to these studies, there are a bunch of scholars who just used BFS as a variable to predict China’s building energy and material demand in the future [7,43,44,45]. Zhou et al. put forward a bottom-up LEAP model and applied it to the energy consumption prediction of the end-use sectors [7]. However, they directly obtained the commercial BFS by deducting residential building floor space (RBFS) from the total BFS listed in *Statistic Yearbook*. The obtained results are the sum of industrial BFS and commercial BFS, which will cause a bias of the prediction of the commercial BFS in China. As for the estimation of commercial BFS, other scholars also used the same problematic data processing method like Zhou and Lin [25,46,47].

In summary, there are some shortages in the existing research. (1) Regarding the estimation of urban residential BFS, some scholars obtained the results by multiplying the urban per capita BFS and the corresponding population, and other scholars obtained the residential BFS data from *CSY*. These data processing methods are problematic due to there exist many defects with the statistical system in China. (2) In terms of the estimation of commercial BFS, the industrial BFS was not separated from commercial BFS in previous studies. They always obtained commercial BFS by subtracting residential BFS from the total BFS. In fact, industrial BFS was still mixed in commercial BFS, and the commercial BFS was overestimated. (3) The universal estimation method for China’s building floor space is lacking and reliable time-series data on BFS are still unavailable to date. (4) Few have systematically studied the existing problems for BFS related statistical indicators in *CSY*, which always led to the unreliability of building stock estimation.

Based on this, this paper attempts to make up for these shortcomings in this field, and the contributions are just as follows. Firstly, this study retrieves statistical indicators concerning BFS with efforts from *CSY* and comprehensively and systematically analyzes the definitions, scopes and existing deficiencies on those indicators. Such a review can provide the worldwide scholars with a deeper insight to understand *CSY.* Secondly, this study proposes a general estimation method for China’s building floor space. Such newly developed method can overcome the defects of inconsistent statistical caliber in *CSY* and previous studies and even provide a practical way of acquiring reliable and robust corrected data on China’s BFS. Finally, this study estimates and derives robust and reliable data series on China’s historical BFS by type (i.e., commercial buildings, urban/rural residential buildings) from 1996 to 2014 using the proposed method. This can fill the data gap in China’s building stock, which is valuable for government officials in terms of energy conservation and planning. The corrected data series lay a good basis for future research on building stock and energy efficiency improvement.

## 3. Systematic Analysis of the Deficiencies for the Items Concerning BFS in *CSY*

Based on the comprehensive review on relevant counting systems related to BFS, three categorizes of statistic indicators regarding BFS can be found in *CSY*: total floor space (year-end), floor space completed this year and per capita floor space of the residential building (PBRFS). In this section, this study will conduct in-depth and systematic analysis on the problems of the BFS related statistical indicators in *CSY*. The following issues are identified when directly obtaining the data on the commercial BFS, urban residential BFS and rural residential BFS from the Statistical Yearbook [11].

### 3.1. Statistical Caliber Inconsistency

**Figure 1:**
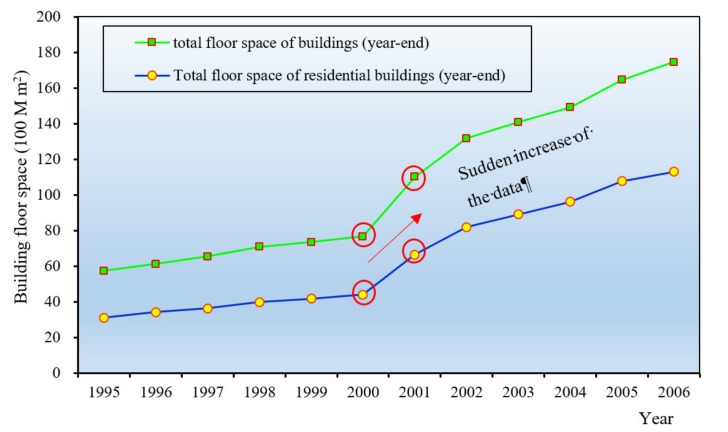
The total floor space of residential buildings (TBFS) and the TRBFS from 1995 to 2006.

There exist substantial changes in the statistical caliber for the total floor space of urban buildings (year-end) (TUBFS). The urban BFS data is mainly from two statistical indicators: total floor space of buildings (TBFS) and the total floor space of residential buildings (TRBFS) (Statistic data is shown in Figure 1). The data related to both two indicators after 2006 were missing in the statistical yearbook. 

As shown in Figure 1, the TBFS and the TRBFS changed suddenly from 2000 to 2001. The reason is that the statistical scope has changed greatly, from the original city scale to the city and county scale. Therefore, to avoid the inconsistency of the statistical range of the BFS, this study will correct the statistics data from 1996 to 2002.

**Figure 2:**
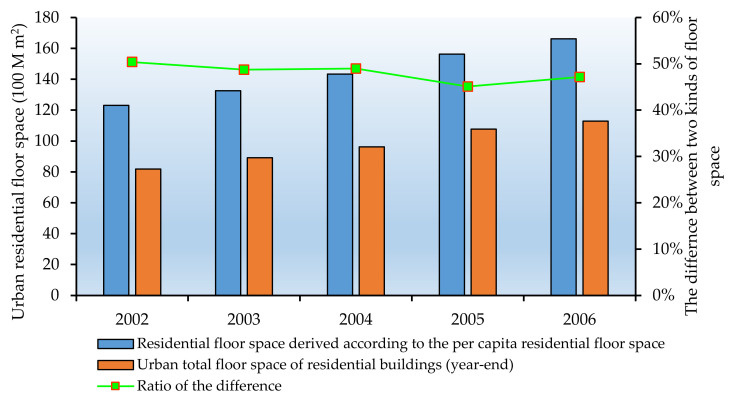
The comparison between the two calibers of the UBRFS.

Currently, the data regarding the urban residential floor space in the statistical yearbook includes TBRFS and PBRFS in urban areas. However, there is a large difference between the TBRFS and the RBFS derived from the per capita floor space. The latter is 40–50% higher than the former, 4 billion m^2^ higher than the former on average, as shown in Figure 2.

As shown in Figure 2. The reasons for such inconsistency are explained as follows. The data source for PBRFS in urban areas is derived from the urban household survey. The urban household survey program is merely performed on the sample households and it needs to carry out daily record work, with the sample updating cycle of 3 years. Therefore, when selecting the sample households, the collective households are excluded and the rental households with high mobility are also be excluded. The living space of the rental households and the collective households are always smaller than those of the private housing groups. This leads to the higher RBFS calculated according to the per capita indicator over the TBRFS.

### 3.2. Data Sources Inconsistency

**Figure 3:**
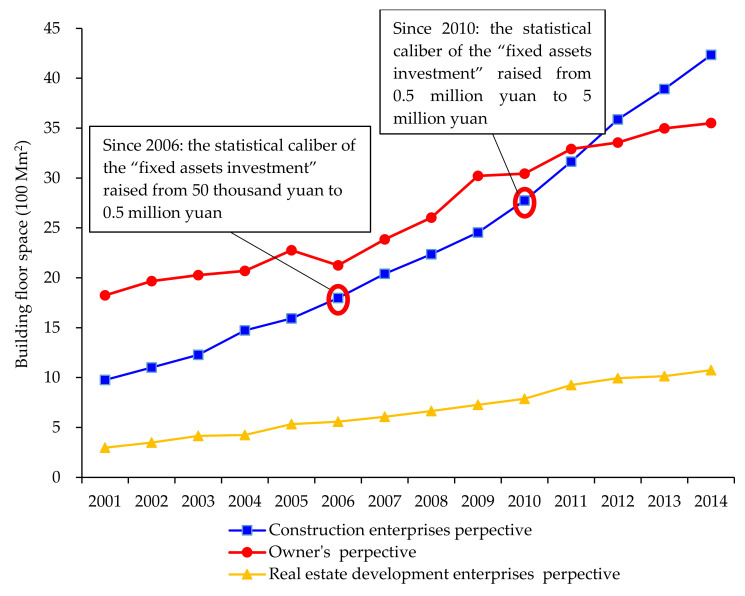
The changing trend of the floor space of buildings completed in different calibers throughout the whole country from 2001 to 2014.

There are three data sources for the “floor space of buildings completed (BFSC)” listed in *CSY*: reporting system from fixed assets, construction industry and real estate. The changing trend of the statistical indicator of the BFS in the above three sources is shown in Figure 3.

In theory, the statistical system based on the owner’s perspective is the most comprehensive one among those statistical systems, and therefore, the calculation of floor space should be based on the “BFSC in the whole country” in the “fixed assets investment (FAI)” part in the Statistical yearbook. However, since the statistical caliber has changed several times in the FAI perspective during 2001–2014, the data in different periods is incomparable and even not suitable for time series analysis. As shown in Figure 3, there are two turning points in the changing trend, which indicate the two major changes for the statistical caliber of the statistical system for “investments in fixed assets”.

Specifically, first, the statistical caliber of the statistical system for “FAI” changed in 2006. Since 2006, the statistical caliber for non-farm household investment in fixed asset statistics has changed to “counting by projects”. The survey method changed from “sample survey” to “comprehensive statistical and report”, and the counting threshold was raised from 50 thousand yuan to 500 thousand yuan. The statistical caliber for the private investment in building construction in urban, industrial and mining areas changed to “counting by projects”, and the starting point for counting was 5 million yuan. Second, the statistical caliber changed in 2011. Since 2011, the statistical starting point for the FAI project was raised from 50 thousand yuan to 500 thousand yuan measured by planned total investment, in addition to real estate development investment and the farm household investment. For the sake of comparison, the China Bureau of Statistics adjusted the corresponding data in 2010 according to the new caliber.

Compared with the “investment in fixed assets”, the statistical caliber for the construction industry does not change significantly, which makes the changing trend of the “BFSC by construction enterprises” more stable and be suitable for time series analysis. In addition, the quarterly data on the construction industry statistics report contains the floor space completed by use, which helps to analyze the building floor space data for different uses. The BFSC from the data source of the “Statistical and reporting system for real estate” is limited to real estate development projects, without including enterprises or individuals’ self-built housing. This smaller statistical range is not suitable for the analysis of the floor space in the whole country. Therefore, this study uses the “BFSC” in the statistical channel of “Statistical and reporting system for the construction industry” as the raw data for estimation in methodology section.

### 3.3. Data Time Series Inconsistency

**Figure 4:**
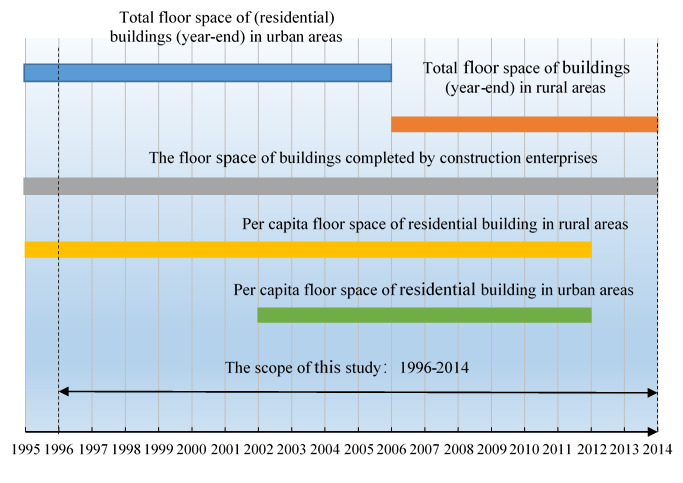
Sketch map of the data loss level of the statistical indicators regarding the building-related floor space.

In terms of the time series of this study (1996–2014), there is data loss in varying degrees on the statistical indicators shown in Figure 4. Specifically, firstly, the data on TUBFS and TUBRFS after 2006 are missing. Secondly, the data on TUBFS after 2006 are missing. Thirdly, the data on PBRFS in urban areas from 1996 to 2001 and from 2013 to 2014 are missing. Finally, the data on PBRFS in rural areas from 2013 to 2014 are missing.

As for the commercial building floor space (CBFS), the data is also lacking. This is due to the commercial floor space being obtained by directly deducting urban RBRFS from the urban RBFS. The industrial building floor space is also included in the derived results [10,11].

## 4. Methodology

### 4.1. Estimation Method for the Rural Residential Floor Space

As discussed above, the per capita residential building floor space in rural areas derived through the rural household survey can objectively reflect the situation of the total amount of buildings in rural areas. Therefore, the rural residential floor space can be calculated according to the rural population and per capita residential building floor space. The calculation formula is shown in the Equation (1):

(1) $$S_{RUR}^{RB}=P_{RUR}\times A_{RUR}$$

where $S_{RUR}^{RB}$ represents rural residential floor space, $P_{RUR}$ denotes the rural population and $A_{RUR}$ represents the per capita rural residential building floor space. The data on both two indicators can be obtained from *CSY* [24,48].

### 4.2. Estimation Method for Urban Residential Floor Space

The TRBFS in urban areas from 1996 to 2006 is available in *CSY* [49]. According to Section 3, the TRBFS changed suddenly from 2000 to 2001, because the Statistical department of China adjusted the statistical scope of those indicators gradually from city-scale to city and county scale since 2001. From the floor space difference between the urban residential stock in year t and the urban residential stock in year t−1, it was found that the changing trend of this floor space difference tended to be stable after 2003. Therefore, the statistics data on the urban residential stock in 2001 and 2002 were still unreliable, since they contained the incomplete adjusted county residential building stock. Those two years’ data will be estimated and corrected respectively by adding the missing county residential building stock.

The urban residential building stock (URBS) from 2003 to 2006 can be calculated by quoting directly the TRBFS in urban areas from 2003 to 2006 in *CSY* [49]. The URBS during 1996–2002 needs to be corrected by adding the missing county level’s residential building stock. The URBS during 2007–2014 can be simulated with the fitted Non-linear Regression (NLR) Model based on the data from 1996 to 2006.

**Step 1: Correct the URBS from 1996 to 2002**

This study assumes that the urban dwelling stock in year t is correlated with the urban dwelling stock in year t−1, the floor space of urban commercial buildings completed in urban areas in year t, demolished residential floor space in urban areas in year t and the floor space increased due to the administrative division in China (The administrative division means that the Township will turn into the Sub-district with the urban sprawl due to the rapid urbanization process in China. In this case, the urban building stock will increase due to this administrative division). Therefore, the following formula can be used to represent the functional relationship among these indicators, which are shown in the Equation (2):

(2) $$S_{URB\left(t\right)}^{RB}=S_{URB\left(t-1\right)}^{RB}+S_{URB\left(t\right)}^{Rcom}-S_{URB\left(t\right)}^{Rdem}+A_{URB\left(t\right)}^{R}$$

where $S_{URB\left(t\right)}^{RB}$ represents the URBS in year t; $S_{URB\left(t-1\right)}^{RB}$ denotes the URBS in year t−1. $S_{URB\left(t\right)}^{Rcom}$ refers to the floor space of residential buildings completed in urban areas of year t. $S_{URB\left(t\right)}^{Rdem}$ represents urban demolished residential BFS in year t, and $A_{URB\left(t\right)}^{R}$ represents the increased urban residential BFS due to the administrative division in China in year t. The “TRBFS in urban areas” and the “floor space of residential buildings completed in urban areas” during 1996–2006 can be obtained directly from *CSY* [49].

The data on the demolition BFS $S_{URB\left(t\right)}^{Rdem}$ is unavailable in *CSY*. Nevertheless, the demolition is related to the floor space started (FSS) (“The floor space started” means the floor space of new buildings that were started to be constructed in a given year), which can be obtained from *CSY*. Therefore, the FSS can be used to estimate the demolition BFS. The FSS in one given year is associated to two parts: (1) the undeveloped land that is used to construct new buildings in this year and (2) the developed land in urban areas with buildings waiting to be demolished in this year. Only when these buildings are demolished, the land can be used to construct new buildings. Therefore, according to the analysis, the demolished BFS is proportional to the FSS to some extent. The coefficient of proportionality is changing over time, which means that there exists a functional relationship between the coefficient of proportionality and time factor T. Therefore, this study uses $f_{R}\left(T\right)\left(T=t-1996\right)$ to represent the coefficient of proportionality of demolished residential BFS on the residential FSS. Therefore, $S_{URB\left(t\right)}^{Rdem}$ equals to $f_{R}\left(T\right)$ multiplied by $S_{URB\left(t\right)}^{Rstart}$. $S_{URB\left(t\right)}^{Rstart}$ represents the residential FSS in year t.

Similarly, the urban residential floor space increased due to the administrative division $A_{URB\left(t\right)}^{R}$ is proportional to the number of Sub-districts to some extent. This coefficient of proportionality is also changing over time, which means that there exists a functional relationship between the coefficient of proportionality and time factor T. Therefore, $g_{R}\left(T\right)$ is used to represent the coefficient of proportionality of the increased URBS due to the administrative division on the number of Sub-districts. $A_{URB\left(t\right)}^{R}$ equals to $g_{R}\left(T\right)$ multiplied by $Sub_{\left(t\right)}$. $Sub_{\left(t\right)}$ represents the number of Sub-districts of year t. The sum of $S_{URB\left(t\right)}^{Rdem}$ and $A_{URB\left(t\right)}^{R}$ can be represented with the following Equation (3):

(3) $$S_{URB\left(t\right)}^{Rdem}+A_{URB\left(t\right)}^{R}=f_{R}\left(T\right)\times S_{URB\left(t\right)}^{Rstart}+g_{R}\left(T\right)\times Sub_{\left(t\right)}$$

Then, this study uses the data on two indicators (i.e., the “urban residential FSS” and the “the number of the Sub-districts”) during 1996–2006 in *CSY* to fit the Function (3). Therefore, this study removes the outlier data on these two indicators in 2001 and 2002 and then uses the remaining data during 1996–2006 to fit the equation with the NLR model. The *R* square of the fitted NLR equation is 0.960, and this can indicate the reliability of the model. Then the estimated data on URBS from 1996 to 2002 can be estimated according to Equation (3).

Next, this study will correct the TRBFS from 1996 to 2002 by adding the missing county scale data. The adjusted county residential building stock $S_{Cou\left(2002\right)}^{RB\left(a\right)}$ in 2002 equals to the statistical data of URBS $S_{URB\left(2002\right)}^{RB\left(s\right)}$ in 2002 deducting estimated URBS $S_{URB\left(2002\right)}^{RB\left(e\right)}$ in 2002. The calculation formula for 2002 is in the Equation (4), and the adjusted county residential building stock $S_{Cou\left(t\right)}^{RB\left(a\right)}$ during 1996–2001 is calculated with the Equation (5).

(4) $$S_{Cou\left(2002\right)}^{RB\left(a\right)}=S_{URB\left(2002\right)}^{RB\left(s\right)}-S_{URB\left(2002\right)}^{RB\left(e\right)}$$

(5) $$S_{Cou\left(t\right)}^{RB\left(a\right)}=\frac{S_{URB\left(t\right)}^{RB\left(e\right)}\times S_{Cou\left(t+1\right)}^{RB\left(a\right)}}{S_{URB\left(t+1\right)}^{RB\left(e\right)}}$$

Through the Equation (6), we can estimate the corrected URBS from 2001 to 2002, and the Equation (7) can be used to estimate the corrected URBS from 1996 to 2000.

(6) $$S_{URB\left(t\right)}^{RB\left(c\right)}=S_{URB\left(t\right)}^{RB\left(e\right)}+S_{Cou\left(t\right)}^{RB\left(a\right)}$$

(7) $$S_{URB\left(t\right)}^{RB\left(c\right)}=S_{URB\left(t\right)}^{RB\left(s\right)}+S_{Cou\left(t\right)}^{RB\left(a\right)}$$

where $S_{URB\left(t\right)}^{RB\left(c\right)}$ represents the corrected URBS in year t. Then, the corrected URBS from 1996 to 2002 can be derived according to the Equations (2)–(7).

**Step 2: Estimate the URBS from 2007 to 2014**

Based on the functional relationship fitted above, the TRBFS in the urban area from 2007 to 2014 can be simulated by inputting the floor space of residential buildings completed during 2007–2014 into Equations (2) and (3).

### 4.3. Estimation Method for Rural Commercial Floor Space

In line with the statistical caliber for urban and rural construction, rural areas contain township built-up areas and villages. In *CSY*, the time series data on rural commercial floor space is available for the period 2006–2015. The changing amplitude of the rural commercial floor space is very little and shows a slow growth trend. The changing scope is roughly between 22 and 25 million m^2^. It is obvious that the data of rural commercial floor space in 2008 is an outlier. Therefore, rural commercial floor space from 2006 to 2015 excluding 2008 can be fitted with a linear regression equation.

(8) $$S_{RUR}^{CB}=\alpha \times t+\beta$$

where $S_{RUR}^{CB}$ represents rural commercial floor space, and t refers to the year. Then, rural commercial floor space from 1999–2005 can be extrapolated according to Equation (8).

### 4.4. Estimation Method for Urban Commercial Floor Space

This section will show how to separate urban commercial floor space from industrial floor space.

**Step 1: Split out the Statistics Data on Urban Commercial Floor Space from 1996 to 2006.**

The sum of the industrial BFS and the commercial BFS in urban areas can be obtained from *CSY*, and the calculation formula is shown in the Equation (9):

(9) $$S_{URB}^{CB}+S_{URB}^{IB}=S^{B}-S^{RB}$$

where $S_{URB}^{CB}$ represents urban commercial floor space, $S_{URB}^{IB}$ represents industrial floor space, $S^{B}$ denotes the TBFS in urban areas, and $S^{RB}$ represents the TBRFS in urban areas. Therefore, this study just needs to identify the ratio of urban commercial floor space and the industrial floor space, and the urban commercial BFS from 1995 to 2006 can then be derived. There exists a relationship between this ratio and the proportion of commercial construction land and industrial construction land, in the Equation (10):

(10) $$\frac{S_{URB}^{CB}}{S_{URB}^{IB}}=\frac{K_{URB}^{CB}L_{URB}^{CB}}{K_{URB}^{IB}L_{URB}^{IB}}=\frac{K_{URB}^{CB}}{K_{URB}^{IB}}\times \frac{L_{URB}^{CB}}{L_{URB}^{IB}}$$

where $S_{URB}^{CB}$ represents the urban commercial floor space, $S_{URB}^{IB}$ represents the urban industrial floor space. $L_{URB}^{CB}$ represents the urban commercial construction land area. $L_{URB}^{IB}$ represents the urban industrial construction area. $K_{URB}^{CB}$ represents the average ratio of commercial BFS on the commercial land, and $K_{URB}^{IB}$ represents the average ratio of industrial BFS on the industrial land. $L_{URB}^{CB}$ and $L_{URB}^{IB}$ can be obtained directly from *China Urban-Rural Construction Statistical Yearbook* [50]. Therefore, only two variables exist in the Equation (10): $K_{URB}^{CB}/K_{URB}^{IB}$ and $L_{URB}^{CB}/L_{URB}^{IB}$. The change of the average commercial BFS on the commercial land (i.e., $S_{URB}^{CB}/L_{URB}^{CB}$) would not show significantly in a short term, and this is the case for the industrial BFS and industrial land. Therefore, we assume the average $K_{URB}^{CB}/K_{URB}^{IB}$ would not experience significant change at the national level in the short term. The validation for the rationality of this assumption is in the appendix. The changing range of $K_{URB}^{CB}/K_{URB}^{IB}$ is not very large. Based on this assumption, we conduct a simulation on $S_{URB}^{CB}/S_{URB}^{IB}$ with MATALAB software to make the changing amplitude of the $K_{URB}^{CB}/K_{URB}^{IB}$ minimum during 1996–2006. We set an initial ratio of urban commercial floor space and industrial floor space (i.e., $S_{URB}^{CB}/S_{URB}^{IB}$), and then, the MATALAB software is used to simulate the dispersion coefficient of the $K_{URB}^{CB}/K_{URB}^{IB}$ minimum. According to Equations (9) and (10), we can split out the statistics data on urban commercial floor space from 1996 to 2006.

**Step 2: Correct the Urban Commercial Building Stock from 1996 to 2002**

As shown in Figure 1, the total floor space of buildings and the TRBFS changed suddenly from 2000 to 2001, due to the statistical scope. This means that the statistical scope of the non-residential BFS (including commercial and industrial BFS) also changed suddenly from 2000 to 2001. Therefore, the correcting method for urban residential BFS is applicable to the correction for urban commercial BFS from 1996 to 2002.

Similar to the correction method for urban dwelling stock, the urban commercial building stock in year t is correlated with the urban commercial building stock in year t−1, the floor space of urban commercial buildings completed in urban areas in year t, demolished the commercial floor space in urban areas in year t and the floor space increased due to the administrative division in year t in China. Therefore, the following formula can be used to represent the functional relationship among these indicators, which are shown the Equation (11):

(11) $$S_{URB\left(t\right)}^{CB}=S_{URB\left(t-1\right)}^{CB}+S_{URB\left(t\right)}^{Ccom}-S_{URB\left(t\right)}^{Cdem}+A_{URB\left(t\right)}^{C}$$

where $S_{URB\left(t\right)}^{CB}$ and $S_{URB\left(t-1\right)}^{CB}$ represent the urban commercial building stock in t and t−1 respectively. $S_{URB\left(t\right)}^{Ccom}$ refers to floor space of commercial buildings completed in urban areas in year t. $S_{URB\left(t\right)}^{Cdem}$ represents urban demolished commercial BFS in year t, and $A_{URB\left(t\right)}^{C}$ represents the increased urban commercial BFS due to the administrative division in China. Like the residential BFS, the sum of $S_{URB\left(t\right)}^{Cdem}$ and $A_{URB\left(t\right)}^{C}$ can be represented with the Equation (12):

(12) $$S_{URB\left(t\right)}^{Cdem}+A_{URB\left(t\right)}^{C}=f_{C}\left(T\right)\times S_{URB\left(t\right)}^{Cstart}+g_{C}\left(T\right)\times Sub_{\left(t\right)}$$

where $S_{URB\left(t\right)}^{Cstart}$ represents the commercial FSS in year t, and $Sub_{\left(t\right)}$ represents the number of Sub-districts in year t. Like correction for urban residential dwelling stock, this study removes the outlier data on those these indicators in 2001 and 2002 and uses the remaining data from 1996 to 2006 in *CSY* to fit the Function (12) with the NLR model. The *R* square of the fitted NLR model equation is 0.899, and the reliability of the proposed model can be ensured. Then, the corrected urban commercial BFS from 1996 to 2002 can be derived adopting the same method like urban BFS (see Formulas (5)–(7)). The urban commercial building stock during 2003–2006 can quote directly the split statistics data of commercial building stock in Step 1.

**Step 3: Estimate the Urban Commercial Building Stock from 2007 to 2014**

Based on the functional relationship fitted above, the total urban commercial building stock during 2007–2014 can be simulated by inputting the floor space of commercial buildings completed in rural areas during 2007–2014 into the Equations (11) and (12).

## 5. Results and Analysis

**Figure 5:**
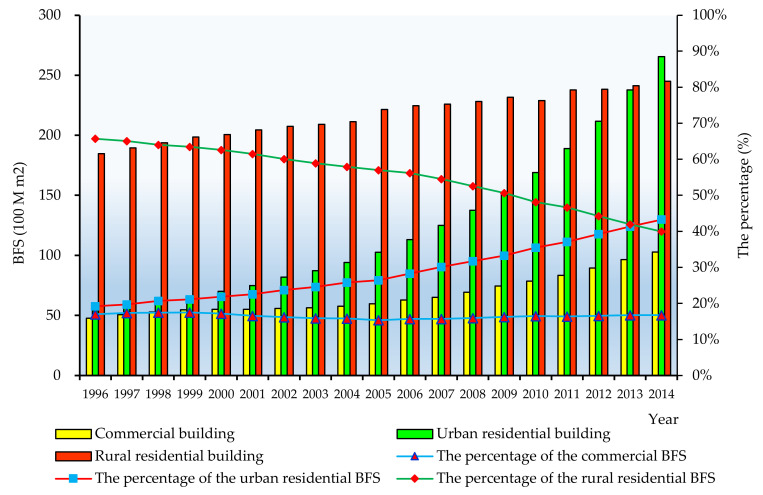
The composition of the building floor space (1996–2014).

According to Equations (1)–(12), the composition of the total BFS, commercial BFS, urban residential BFS and rural residential BFS can be derived, which is shown in Figure 5.

As Figure 5 shows, the total floor space of China witnessed an upward trend from 28.1 billion m^2^ in 1996 to 61.3 billion m^2^ in 2014, more than twofold increasing during the covered period. The average increase rate of the BFS was 4.4% annually during 1996–2014.

### 5.1. Characteristics of China’s Building Floor Space

From Figure 5 we can see the urban residential BFS increased 21.2 billion m^2^ from 1996 to 2014, and rural residential BFS only grew by 6 billion m^2^. This indicated the rapid urbanization of China in recent years. The proportion of the urban residential floor space grew more than two times from 19.2% in 1996 to 43.3% in 2014, whereas the figure for the rural residential floor space declined correspondingly from 65.7% to 40.0% from 1996 to 2014. In 2014, the urban residential BFS overtook the rural residential floor space and accounted for the largest proportion in the BFS in China. During 1996 to 2014, the commercial floor space increased more than tripled, from 4.8 billion m^2^ to 10.3 billion m^2^, although its percentage was only 16%.

**Figure 6:**
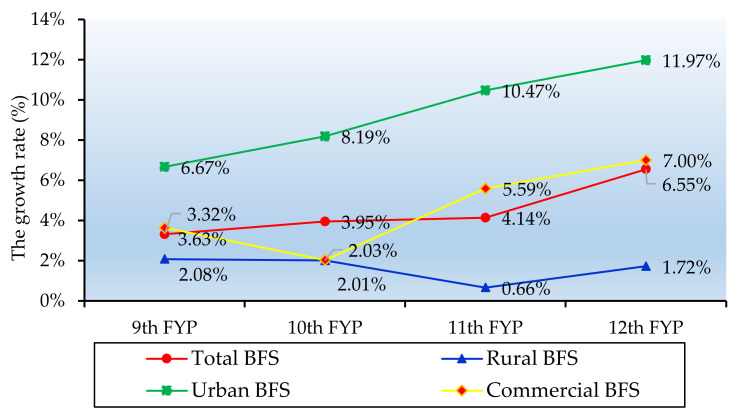
The composition of the building floor space (1996–2014).

According to the results demonstrated in Figure 5, the changing trend of the growth rate of China’s buildings floor space by type is shown in Figure 6.

Moreover, from Figure 6, we can see that the whole growth rate of the total BFS during the “12th Five Year Plan (FYP)” period was the highest (i.e., 6.6%), which is almost twice the figure during the “9th FYP” period (i.e., 3.3%). From the period of 1996–2014, the urban residential BFS witnessed higher increase rate than the other types of BFS, and it showed a sustained growth. The rate of increase of the commercial BFS showed a sharp increase during the whole period, increasing from 3.6% during the “9th FYP” period to 7.0% during the “12th FYP” period. The rural residential BFS grew the least compared to the commercial urban residential BFS, with a figure of less than 2% during the “11th FYP” and the “12th FYP” period. As for the average annual increase rate, three types of BFS sawed different figures: 9.3% for the urban residential BFS, 4.4% for the commercial BFS and 1.6% for rural residential BFS.

### 5.2. Residential Building Floor Space

**Figure 7:**
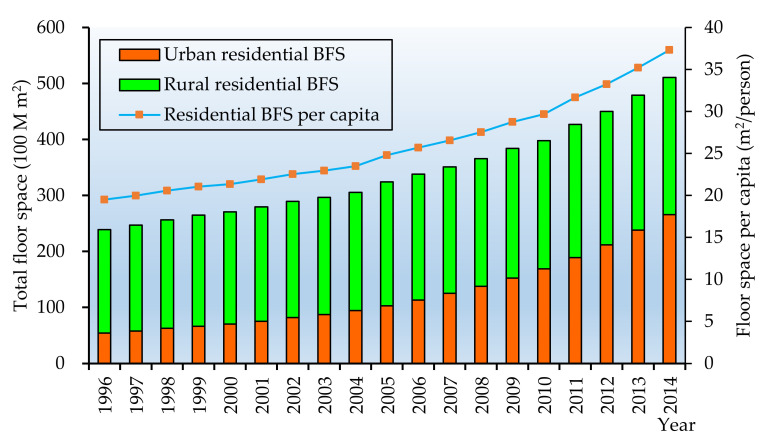
The residential BFS and per capita residential floor space (1996–2014).

Figure 7 shows the residential BFS and per capita residential BFS in China throughout 1996–2014.

From Figure 7, we can see that the total residential floor space grew significantly by 26.1 billion m^2^ from 1996 to 2014. The average increase rate was 4.3% annually. The per capita residential floor space increased almost two times from 19.5 m^2^/person to 37.3 m^2^/person over the period of 1996–2014, which is due to the decline of the household size and the growth of the middle class given the higher living standard. This is one of the drivers for the residential BFS dramatic increase. Another reason is the urbanization rate. In line with the data on *CSY*, the urbanization rate has increased from 30.5% in 1996 to 59.5% in 2019 and is expected up to 80% by 2050 [13]. In order to accommodate the increasing new migrants from the rural area to the urban area, more residential buildings were constructed.

### 5.3. Commercial Building Floor Space

**Figure 8:**
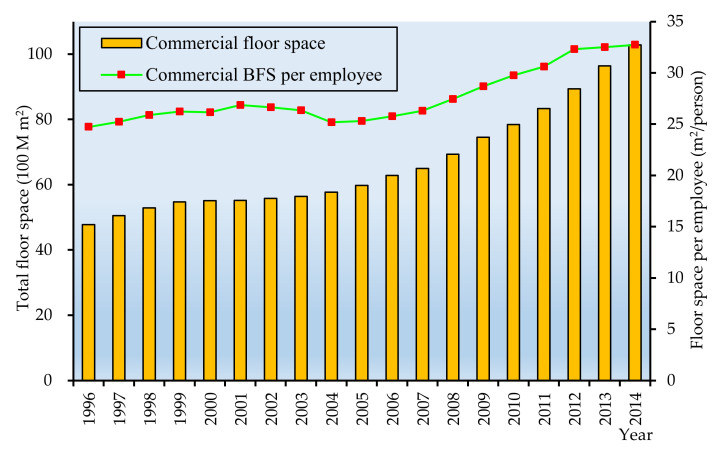
The commercial building floor space (BFS) and commercial floor space per employee (1996–2014).

The commercial BFS and the commercial BFS per employee (The people in the commercial buildings are mainly employed laborers in the tertiary sector (i.e., the service sector). Therefore, the commercial BFS per capita is actually the commercial BFS per employee) in China over the period of 1996–2014 are shown in Figure 8.

From Figure 8, we can see that the total commercial floor space increased dramatically by 5.5 billion m^2^ during 1996–2014. The average increase rate was 4.4%. The possible reason maybe the increase of the commercial BFS per employee and the growth of the service sector employment share. As shown in Figure 8, the commercial floor space per employee increased from 24.7 m^2^/employee to 32.7 m^2^/employee over the period of 1996–2014. Based on the data released in *CSY*, the percentage of the tertiary industry employee witnessed a sharp increase, from 26.0% to 40.6% during the period 1996–2014. The root cause is that a large proportion of migrant workers moved to urban areas with the rapid urbanization process in recent years, and most of them worked in the manufacturing and service sectors, approximately 70% and 22%, respectively, in 2010 [51,52].

## 6. Reliability Analysis and Discussions

### 6.1. Reliability Analysis for Urban Residential and Commercial Building Floor Space

**Figure 9:**
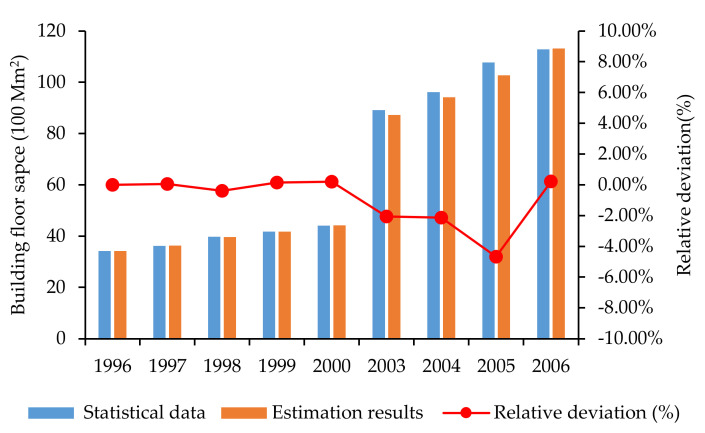
The deviations between the model results and the statistical data as for urban residential floor space from 1996 to 2006 excluding 2001 and 2002.

In terms of the estimation of the urban residential BFS, the study adopted the NLR model to fit a nonlinear equation with data on the “urban residential floor space”, the “floor space of residential buildings completed”, “residential FSS” and “the number of the Sub-districts” for the period 1996–2006. The deviations between the model results and the statistical data as for the urban residential floor space from 1996 to 2006 are shown in Figure 9.

From Figure 9 we can see, the relative average deviation between the model results and the statistical data regarding urban residential floor space is 1.08%, the minimum deviation is −4.67%, and the maximum deviation is 0.22%. This indicates the good fitness and validity of the NLR model can be verified. Therefore, the reliability of the data on the urban residential floor space from 2007 to 2014 estimated by the NLR model can be ensured.

For the estimation of the urban commercial BFS, the study adopted the NLR model to predict urban commercial floor space during 2007–2014 with data on the accumulated commercial BFS completed during the same period. To verify the validity and reliability of the proposed model, the model result and the statistical data from 1996 to 2006 need to be compared. The deviations between the model results and the statistical data from 1996 to 2006 are shown in Figure 9. The minimum deviation is −1.41%, the maximum deviation is 1.57%, and the maximum relative deviation is less than 5%, which can verify the quality of the model and robustness of the results.

### 6.2. Reliability Analysis for Rural Residential and Commercial Building Floor Space

As discussed in the methodology, the rural residential BFS equals the total of the rural population multiplied by PBRFS.

Unlike urban residential buildings, rural residential buildings are not related to the collective residents. The historical raw data are all based on statistical data of good quality, so the estimation of the rural residential BFS is of low uncertainty.

**Figure 10:**
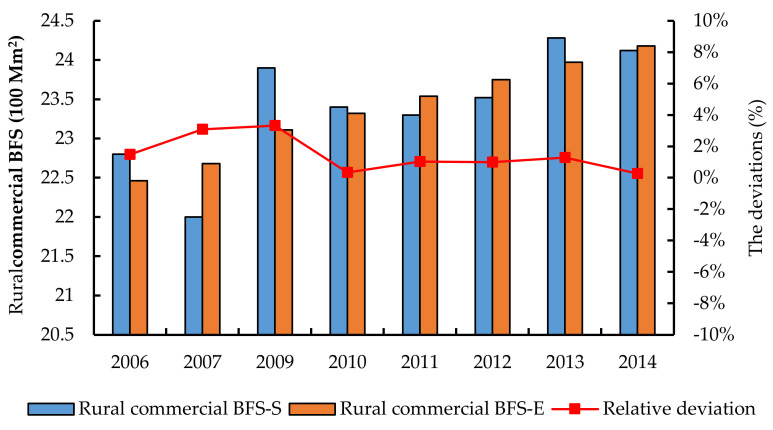
Comparison between the model results and the statistical data as for rural commercial floor space from 2006 to 2014 excluding 2008.

As described in the methodology, the changing amplitude of the rural commercial floor space during 2006–2015 was very small and showed a slow-growth trend. The deviation of the linear regression model is shown in Figure 10. It is found that the maximum and minimum relative deviation is 3.32% and 0.33%, respectively. This can verify the reliability of the model result.

The statistical data is released by the official authority, China Statistics Bureau. However, there exists some deficiencies as the data as mentioned in Section 3. That is why we propose a set method to estimate the data. Our estimated data was close to the statistical data, which meant that our methods were reliable and could obtain reliable results.

### 6.3. Discussion

As it is known, the Chinese government is emphasizing the development of urbanization. During the “12th FYP” period, urbanization was viewed as the powerful engine to enhance strength for turning economic growth in an environmentally friendly direction. It is obvious that the rapid growth of the urban residential BFS contributed the most to the increase rate of the total BFS in China throughout the whole period. The rapid urbanization of China during 1996–2014 may account for the sharp increase of the urban residential BFS. The possible causes for the increase of the commercial building floor space are the urbanization process and the economic development. With the urbanization process, a large proportion of migrant workers moved to urban areas to seek jobs, and most of them are occupied in service sector related work. This would promote economic activity in the service sector, which would result in an increase in commercial building floor space demand and commercial building energy consumption.

## 7. Conclusions

This study comprehensively discussed the BFS related indicators in *CSY* in detail and proposed a general estimation method aiming at the defects in *CSY* and previous studies. Then the BFS in China by type during 1996–2014 were estimated using the proposed model. Our research results can be useful for the government and decision makers to set effective building stock plans and building energy-efficiency policies and measures to address the increasing building energy consumption and carbon emissions. The main findings are as follows:

1. This study comprehensively analyzed the existing deficiencies regarding the BFS related statistical indicators in *CSY* and found that: (1) The statistical caliber on the TUBFS changed over time. (2) The floor space of completed suffers from a heterogeneous data source with great discrepancies. (3) Most of the statistical indicators suffer from incomplete time series data. (4) The data regarding urban commercial floor space cannot be obtained in *CSY*. (5) For urban residential building area, there is a big gap between the two statistical calibers.
2. China BFS were estimated adopting the proposed method, and results indicated that China BFS was 61.3 billion m^2^ in 2014. Of this, commercial, urban residential and rural residential BFS were 10.3 billion m^2^, 26.5 billion m^2^ and 24.5 billion m^2^, respectively. Of China’s BFS in 2014, the urban residential BFS accounted for the largest proportion, 43.3%, like the percentage of the rural residential (40.0%), and the figure for the commercial building was the lowest, 16.8%.
3. The total floor space in China witnessed an upward trend and increased by 33.2 billion m^2^ from 1996 to 2014, increasing about two times during this period. The average increase rate of the BFS in the whole period was 4.4% annually. During the period 1996–2014, three types of BFS saw various growth rates: 9.3% for the urban residential BFS and 4.4% and 1.6% for commercial and rural residential BFS, respectively. The rapid growth of the urban residential BFS contributed the most to the increase rate of the total BFS in China throughout the whole period.
4. By comparing with the statistical data, we found that the deviations were well below 5%. This could indicate the reliability of the results and robustness of the proposed method.

In the future, we will employ a dynamic stock model to examine the dynamic change of Chinese BFS cohorts, type, time, etc. Moreover, the dynamic change of retrofit buildings, demolished buildings and future buildings will also be examined in the next study.
